# Multiplex CRISPR/Cas9-mediated genome editing to address drought tolerance in wheat

**DOI:** 10.1080/21645698.2022.2120313

**Published:** 2022-10-06

**Authors:** Naglaa A. Abdallah, Hany Elsharawy, Hamiss A. Abulela, Roger Thilmony, Abdelhadi A. Abdelhadi, Nagwa I. Elarabi

**Affiliations:** a Department of Genetics,Faculty of Agriculture, Cairo University, Giza, Egypt; b Chemistry Department, Faculty of Science, Cairo University, Giza, Egypt; c USDA-ARS Crop Improvement and Genetics Unit, Albany, California, USA

**Keywords:** CRISPR-Cas9, drought tolerance, *Sal1* gene, stomata, wheat

## Abstract

Genome editing tools have rapidly been adopted by plant scientists for crop improvement. Genome editing using a multiplex sgRNA-CRISPR/Cas9 genome editing system is a useful technique for crop improvement in monocot species. In this study, we utilized precise gene editing techniques to generate wheat 3’(2’), 5’-bisphosphate nucleotidase (*TaSal1*) mutants using a multiplex sgRNA-CRISPR/Cas9 genome editing system. Five active *TaSal1* homologous genes were found in the genome of Giza168 in addition to another apparently inactive gene on chromosome 4A. Three gRNAs were designed and used to target exons 4, 5 and 7 of the five wheat *TaSal1* genes. Among the 120 Giza168 transgenic plants, 41 lines exhibited mutations and produced heritable *TaSal1* mutations in the M_1_ progeny and 5 lines were full 5 gene knock-outs. These mutant plants exhibit a rolled-leaf phenotype in young leaves and bended stems, but there were no significant changes in the internode length and width, leaf morphology, and stem shape. Anatomical and scanning electron microscope studies of the young leaves of mutated *TaSal1* lines showed closed stomata, increased stomata width and increase in the size of the bulliform cells. *Sal1* mutant seedlings germinated and grew better on media containing polyethylene glycol than wildtype seedlings. Our results indicate that the application of the multiplex sgRNA-CRISPR/Cas9 genome editing is efficient tool for mutating more multiple TaSal1 loci in hexaploid wheat.

## Introduction

Global climate change is one of the main challenges for agricultural production by creating problems for farmers throughout the world and exacerbating or in some cases causing abiotic stress conditions that affects food production. Crop productivity worldwide is seriously affected by drought causing economic losses. Developing abiotic stress tolerant crops, like wheat, will help mitigating climate change challenges and help in ending hunger. Wheat (*Triticum aestivum*) is the most important staple food crops world-wide. It provides more than 30% of all calories for the world population, and it is the main source of processing bread, cookies, and noodles.^1^ It is expected that wheat production will face unprecedented challenges due to the global climate change, increasing world population, and water shortages in arid and semi-arid lands, however, the increasing in its productivity during the last decade do not cover the population consumption. As irrigation water sources have become scarcer, many breeding programs target the development of drought tolerant crop cultivars. Moreover, the hexaploid (2 n = 6x = 42, AABBDD) nature, and gene functional redundancy of wheat make the genetics improvement complicated and time-consuming to obtain the desired phenotype.^2^ To ensure global food and ecosystem security, it is essential to enhance the flexibility of wheat production through the use of cutting-edge technologies.^3^

Clustered regularly interspaced short palindromic repeat (CRISPR)-CRISPR-associated protein (CRISPR-Cas) system, is recently used as a simple, accurate and inexpensive system for precise, sequence-specific modifications of DNA sequences. Therefore, it is highly acceptable over other genome editing techniques toward improvement of the desired traits in crops such as management of abiotic and biotic stresses, nutrient improvement, and breeding improvement many crops.^4,5^ Genome editing could be used to induce gene knockout, single-base substitution, and gene/allele replacement *in vivo*. Genome sequence screening using in silico tools is required for obtaining optimal design of gRNA and Cas expression constructs as well as a suitable transformation and regeneration systems for delivering the transgenes, proper mutation screening. The DNA breaks generated by CRISPR/ Cas, are repaired in the cell by the non-homologous end joining (NHEJ) pathway, homology-directed repair (HDR), or both NHEJ and HDR.^6,7^

Due to plant genome complexity and redundancy, alteration in cellular functions may require the alteration of multiple mutations. CRISPR/Cas system could edit multiple genes through gRNA cassettes designed using one or many promoters into a single vector system.^8,9^ Expression of several gRNA cassette could be constructing arrays of gRNA interrupted by tRNA sequence, gRNAs get released with the incision created by endogenous RNase.^10^ In *Arabidopsis thaliana*, researchers used multiple genomes targeting with three gRNA expression cassettes to mutate four subunits of keratin p80, resulting in short statured phenotype in quadruple mutant plants.^11^ Cas9 system has been established for use in *Arabidopsis thaliana*, rice (*Oryza sativa), Nicotiana benthamiana* and wheat (*Triticum aestivum*).^12,13^ In addition, the CRISPR/Cas system has been effectively employed to understand the molecular basis of abiotic stress tolerance in crops. Increasing knowledge regarding the molecular mechanisms underlying abiotic stress responses in crops and advancements in the CRISPR/Cas system have provided new opportunities to generate climate-resilient crops.^14^

The highly compartmentalized cells in plants dependent on the flow of information to and from the nuclei to other compartments of the cell to coordinate their intracellular functions. The monophosphate 3′-phosphoadenosine 5′phosphate (PAP), which regulate the expression of many nuclear genes that affects response to biotic and abiotic stresses.^15^ The gene *Sal1* encodes the enzyme 3’(2’),5’-bisphosphate nucleotidase that catalyzes the conversion of adenosine 3’,5’-bisphosphate (PAP) into adenosine monophosphate and inorganic phosphate.^16^ Sal1 is a dephosphorylating enzyme with dual activities in vitro which are 3’(2’),5’-bisphosphate nucleotidase and inositol polyphosphate-1-phosphatase.^17^ Based on *in vitro* assays of recombinant proteins, two potential substrates for Sal1 have been identified: 3’-phosphoadenosine 5ʹphosphate (PAP), a by-product of sulfate assimilation pathway in plants^18^ and inositol 1,4,5-triphosphate (IP3), a second messenger molecules involved in calcium signaling.^19^ In *Arabidopsis, Sal1* was found to be involved in the drought tolerant mechanism. Previous study showed that *Sal1* acts as a negative regulator of drought tolerance in *Arabidopsis*.^20^ The CRISPR system, a sophisticated and efficient tool for generating precise genomic modifications, was used to inactivate the *Sal1* genes in *Arabidopsis*, tobacco, sorghum, and rice.^21^ Previous research has demonstrated that *sal1* mutant plants (other than wheat) produce protectant stress compounds that enhance drought tolerance.^22^ In this work, we used CRISPR-Cas9-based editing system to create mutations in the *TaSal1* homeform gene family in *Triticum aestivum* cv, Giza168 to study their effects on improve levels of drought stress tolerance in wheat.

## Results

### Construction of Wheat Sal1 CRISPR Based Vectors:

**Figure 1.**
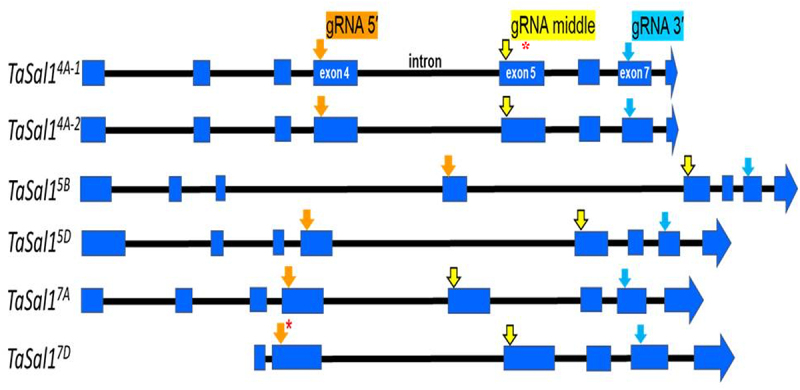
A diagram of the six *TaSal1* genes present in Giza-168 hexaploid wheat showing the exons regions (blue boxes) and the 5’ (Orange arrow) middle (yellow arrow) and 3’ (blue arrow) locations of the gRNA. The * in TaSal1^4A-1^ represents the location of the extra C inserted into the coding region downstream of the middle target sequence, * in TaSal1^7D^ represents single nucleotide mismatch (C instead of T) at the sgRNA.

Based on the *Sal1 Arabidopsis* gene, six homologous *TaSal1* sequences were identified in the Chinese Spring wheat genome (4A, 7A, 5A, 5B, 5D & 7D). An additional gene was found on chromosome 4A of other cultivars as a tandem duplication (i.e. 4A1 and 4A2). Primers designed for the seven putative *TaSal1* genes, were used to amplify and identify the *TaSal1* genes in the Giza 168 genome. All reactions produced bands of the expected size except for 5A specific primers, which failed to amplify any product. This indicates that Giza-168 has likely lost the 5A gene. The amplified hexaploid wheat *TaSal1* sequences were cloned, sequenced, and annotated. The amplified sequences for 4A-1, 4A-2, 7A, 5B, 5D & 7D were sequenced and submitted to the GenBank under accession numbers from ON332560 to ON332565, respectively. The sequence of the PCR products was aligned with the other available wheat genomes with almost all sequences being identical with only a few single nucleotide polymorphisms (SNPs) observed. The only SNP that may be functional change is a single nucleotide insertion in the Giza-168 *TaSal1* 4A-1 sequence, which has an extra C inserted into the coding region. This sequence change results in a frameshift and the insertion of a premature stop codon making this gene likely a nonfunctional allele. From the aligned sequence the intron/exon structure for these six genes, three targeting sequences present were chosen for use as gRNA target sites for gene editing (Fig. 1).

The three designed gRNA sequences were synthesized as oligos and assembled into a single gene with a tandem array of tRNA-gRNA architecture and cloned into an expression cassette under the control of the *PvUbi1* promoter and nopaline synthase terminator (Figure S1). The developed pCas2143 carrying the assembled tRNA-gRNA was verified using the gRNA-specific primers (Table S2). In vivo, the excision of tRNA from the transcript by the endogenous RNases should release the three gRNAs.

### Transformation and Screening of Transformed Plants

**Figure 2.**
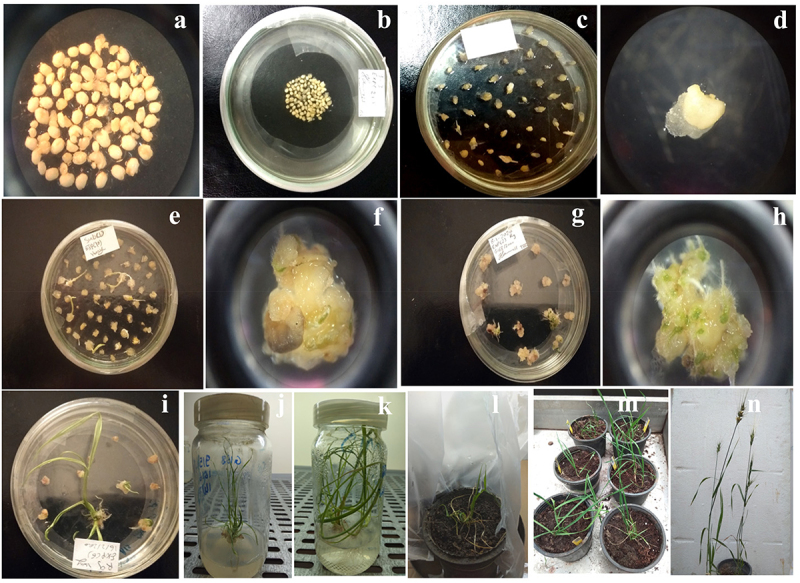
Giza-168 wheat transformation and regeneration stage using immature embryos. A & B: isolated embryos; C, D, E & F: callus formation on callus induction media; G and H: shoot formation on shoot induction media; I and J: shoot elongation and multiplication; K: root formation on root induction media; L and M: acclimatization of putative transgenic plant and N: putative transgenic plant maturation and seed formation.

Two different types of wheat Giza168 explants (mature and immature embryos) were used in this study. Mature and immature embryos isolated from wheat Giza168 was bombarded with a mixture of pAHC20 and pCas2143 constructed vectors using the biolistic gene gun. Putative transgenic calli derived from both mature and immature embryos were regenerated within 2–3 weeks on the MS medium containing 2% sucrose, 0.15 mg/l thidiazuron, and 1 mg/l bialaphos. During the selection process, successfully transformed calli continued to grow vigorously on selection media supplemented with 1 mg/l bialaphos (for selection) to produce shoot initiations. However, the untransformed ones failed to form shoots. From this experiment, about 21700 embryos (20000 immature and 1700 mature embryos) were isolated and bombarded with biolistic gene gun. The transformed embryos produced 18410 calli and 16135 shoots. The shoots were transferred to MS medium supplemented with 2 mg/l BA and developed to 11047 regenerated plant lines (Table 1). The regenerated plants were transferred to MS supplemented with 2.5 mg/L IBA and 2 mg/l bialaphos for root induction. For acclimatization, transformed and rooted plantlets were planted in 8 cm pots containing Hoagland solution. After 1 month in the greenhouse, the plants were transferred to larger pots containing 1:1 (v:v) mixture of peat and perlite and irrigated with tap water (Fig. 2). The regeneration percentage of recovered plants was only 51% (11047 out of 21170) due to acclimatization the recovered plant in inappropriate agricultural season this led to the loss of many plants.

**Table 1.** Regeneration, transformation and mutation efficiency originated from Giza 168 mature and immature embryos using pCas2143 in combination with the pAHC20 plasmids.

Type of explant	Embryos No.	Calli No.	Shoot No.	Regenerated plants No.	Transformed No. (PCR positive)	Transformation %	No. of partial mutated CRISPR lines	No. of full mutated CRISPR lines
Mature	1700	1388	1198	897	4	0.45	4	0
Immature	20000	17022	14937	10150	116	1.14	37	5
Total	21700	18410	16135	11047	120	1.08	41	5

Genomic PCR screening for the *Cas9* gene for all herbicide resistance plants developed from transformed candidates identified 120 PCR positive (1.08%) wheat lines with clear bands corresponding to the expected sizes of 142 and 620 bp for the *Cas9* and *gRNA* specific sequences, respectively (Figure S2). Transgenic (mutated) plants were allowed to grow in biocontainment greenhouse to produce M1 and M2 seeds. The low transformation frequency could be attributed to the usage of co-transformation (two vectors).^23^

### TaSal1 *Mutation Screening Using CAPS and Sequencing*

M_1_ develop the process for screening the transformed Giza-168 wheat plants for CRISPR-induced mutations in the *TaSal1* target genes, wild-type genomic DNA was isolated and used for PCR to amplify the five *TaSal1* genes. Using this screening method, 41 independent M_1_ lines were identified as exhibiting active CRISPR-mediated gene editing. Among them, only five lines (GEL2, GEL19, GEL35, GEL79 and GEL119) have the five mutated *TaSal1* homeoform genes (Table 2), while 36 lines were partially mutated in one or more mutated *TaSal1* gene. The genotypes of these progeny plants were evaluated using genomic PCR both for the introduced transgenes as well as mutations at the *TaSal1* target sites. The mutations in these plants were identified using genomic PCR amplification of the *Sal1* target regions including the CAPS screening assay, and the sequencing of the *Sal1* amplicons. (Example of the PCR results of mutation detection Figure S3).

**Table 2.** Detailed description of the CRISPR-Cas9 *TaSal1* mutations within the wheat edited lines plant.

TaSal1 location	Mutated lines	5’ sequence	Middle sequence	3’ sequence	Mutation Description
gRNA	Control	GGACTCAGAAGACTTGAGAA	GCACTGGCACTGCTTGATGA	AATAGATAGCCAAGCAAAATA	
4A2	GEL2	GGA – – – – –GAA	GCACTGGCAC – – ATG	AATAGA – – – –AATA	14 nt Del at 5’; 6 nt Del at middle; 11 nt Del at 3’
	GEL19	GGA – –CTCAGAAGAGAA	– – – – – – – – – – -ATG	AATAGATAGCCAAGCAATAGCAATA	5 Del at 5’; 31 nt Del at middle; 4 nt Ins at 3’
	GEL35	GGACTCAGAAGACTGGCCATGCATTTGCCTAGAA	GCACTGCTTGGTTTGCATG	AATAGATAGCCAAG – AATA	16 nt Ins at 5’; 6 nt Ins at middle; 3 nt Del at 3’
	GEL79	GGACTCAGAAGACGACTAGAA	GCACTGCTTGATGGCCCATAGTG	AATAGATAGCCAAGC – ATA	4 nt Ins at 5’; 10 nt Ins at middle; 3 nt Del at 3’
	GEL119	GGA – – – – -AGAA	GCA – – – – -ATG	AATAGATAGCCAAGCAA-ATA	13 nt Del at 5’; 13 nt Del at middle; 1 nt Del at 3’
7A	GEL2	GGA – – – – –GAA	– – – – – – –ATGA	AATAGATAGCCAAGCAAGAATA	14 nt Del at 5’; 20 nt Del at middle; 1 nt Ins at 3’
	GEL19	GGACTCAGAAGACTTGTACCCGTCAAATGTCAGAA	GCACTGCTTATTTACCGATGA	AATAGATAGCCAAG – AATA	15 nt Ins at 5’; 7 nt Ins at middle; 3 nt Del at 3’
	GEL35	GGACT – – – –AGAA	GCAC – – – – ATGAAGG	AATA – – – – -AATA	11 nt Del at 5’; 12 nt Del at middle; 13 nt Del at 3’
	GEL79	GGACTCAGAAGA – -AGAA	GCAC – – – – ATGA	AATA – – – – -AATA	4 nt Del at 5’; 12 nt Del at middle; 13 nt Del at 3’
	GEL119	GGACTCAGAAGACTT-AGAA	GCATGGCACTGCTTCCCGATGA	AATAGATAGCCAAGCAACCATATGGAAATA	1 nt Del at 5’;3 nt Ins at middle; 9 nt Ins at 3’
5B	GEL2	NA	GCATGGCACTGCT – TGA	AATAGATAGCCAAGCAAA-TA	3 nt Del at middle; 1 nt Del at 3’
	GEL19	NA	GCATGGCACTGCTTGAAACCTTGA	AATAGATAGCCAAGCAACCTGTCAATA	5 nt Ins at middle; 6 nt Ins at 3’
	GEL35	NA	G – – – – – ATGA	AATAGATAGCCAA – -AATA	15 nt Del at middle; 4 nt Del at 3’
	GEL79	NA	GCACTGGCACTGCT–ATGA	– – – – – – – – –AATA	2 nt Del at middle; 26 nt Del at 3’
	GEL119	NA	GCACTGGCACTGCTTGAATGA	AATAGATAGCCAAGC-AAATA	1 nt Ins at middle; 1 nt Del at 3’
5D	GEL2	GGACTCAGA – – –GAA	GCACTGGCACTGCTT-ATGA	AATAGATAGCCAAGCAATAATA	8 nt Del at 5’; 1 nt Del at middle; 1 nt Ins at 3’
	GEL19	GGACTCAGAAGACTT-AGAA	– – – – – – – – – -ATGA	AATAGATAGCC – –AAATA	1 nt Del at 5’; 28 nt Del at middle; 5 nt Del at 3’
	GEL35	GGACTCAGAAGACTTGTAGAA	GCACTGG – – – ATGA	AATAGATAGCCAAGCAAATTCATA	1 nt Ins at 5’; 9 nt Del at middle; 3 nt Ins at 3’
	GEL79	GGACTCAGAAGAC – AGAA	GCACTGGCACTGCT–ATGA	AATAGATA – – – AATA	3 nt Ins at5’; 2 nt Del at middle; 9 nt Del at 3’
	GEL119	GGACTCAGAAGACTTGTCCAAGAA	GCACTG – – – -ATGA	AATAGATA – – – AATA	4 nt Ins at5’; 10 nt Del at middle; 9 nt Del at 3’
7D	GEL2	GGACTCAGAA – – AGAA	GCACTG – – – -ATGA	AATAGATAGCCAA – -AATA	6 nt Del at 5’; 10 nt Del at middle; 4 nt Del at 3’
	GEL19	GGACTCAGA – – –GAA	GCACTGGCACTGCT–ATGA	– – – – – – – – AATA	8 nt Del at 5’; 2 nt Del at middle; 24 nt Del at 3’
	GEL35	GGACTCAGAAGACTTGCCGTATGCCAGAGAA	GCACTGGCACTGCCTGAACTGTATATATGA	AATAGATAGC – – -AATA	11 nt Ins at 5’; 10 nt Ins at middle; 7 nt Del at 3’
	GEL79	GGAC – – – – AGAA	GCA – – – – -ATGA	AATAGATAGCCAAGCAACCCAATA	12 nt Del at 5’; 13 nt Del at middle; 3 nt Ins at 3’
	GEL119	GGACTCAGAAGAC – AGAA	GCACTGGCAC – – ATGA	AATAGATA – – – AATA	3 nt Del at 5’; 6 nt Del at middle; 9 nt Del at 3’

The 3ʹCRISPR target sites in all 5 of the *TaSal1* gene targets (in Giza-168) contain a native *Xcm*I restriction enzyme recognition site. The PCR amplification for the 3 target sites of the each of the 5 *TaSal1* genes were used with all 120 of the T_1_ Giza168 transgenic lines, using primers specific for each site (Table S4). Developed PCR (Figure S3) were used for CAPS screening assay, *Xcm*I restriction enzyme recognition the 3ʹCRISPR target sites. The restriction enzyme digested nonmutated PCR amplicons into two smaller fragments but was unable to digest amplicons with CRISPR induced InDels in the target region. Out of 120 transgenic lines that exhibit the Cas9 gene, only five lines (GEL2, GEL19, GEL35, GEL79 and GEL119) exhibited a block in *Xcm*I digestion were identified as active CRISPR edited lines. In the mutated lines, the regions surrounding the three target sites (5ʹCRISPR, middle and 3ʹCRISPR target sites) were subjected for nucleotide sequences to identify any kind of *TaSal1* mutations in them.

### *Nucleotide Sequences of the Amplified Products Targeted* TaSal1 *CRISPR Sited*

Sequence results from the GEL 2, GEL 19, GEL 35, GEL 79 and GEL 119 lines show numerous mutations in the *TaSal1* the target regions of the homeoform genes including small deletions and insertions between target sites. A summary of the mutations that have been identified in the progeny from these five lines are shown in Table 2. All detected InDel started at 3–5 nt upstream of the PAM sequence as expected and ranged between 1 and 28 nt. Some mutations included InDel of three or multiplications of three nucleotides that will not cause frameshift or stop codon, but the other target sites in the same genes included other kind of mutations that will block the gene activities. The overall mutations within the *TaSal1* five homeoform genes of the five lines appear to have knock-out the target genes and were unable to produce a functional TaSal1 protein. Thus, the M_2_ progenies of these 5 lines were further evaluated to study the effect of the ‘knock-outs’ *TaSal1* on plant behavior.

NA = not applicable

### Morphological Studies

**Figure 3.**
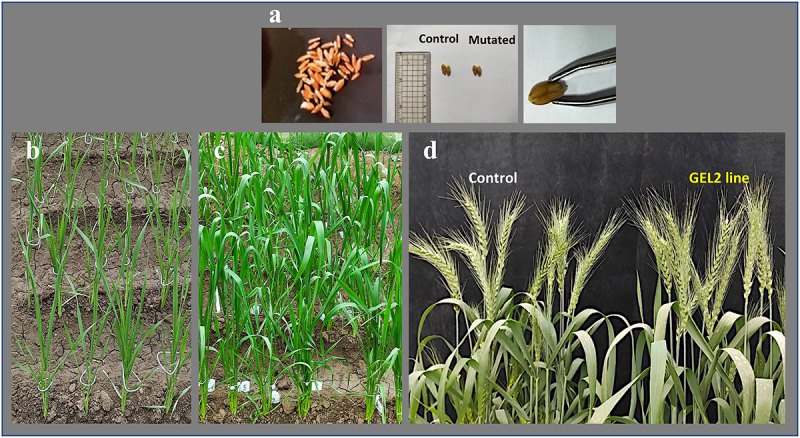
Stages of maturity of well-watered *TaSal1* mutant Giza 168 A: Seeds obtained from transgenic plants (T_0_), B: Status of the plants 37 days post germination, C: 60 days post germination, D: Wheat plants developing spikes harvested from wildtype Giza168 and GEL2 mutant plants grown in the same well-watered greenhouse.

**Figure 4.**
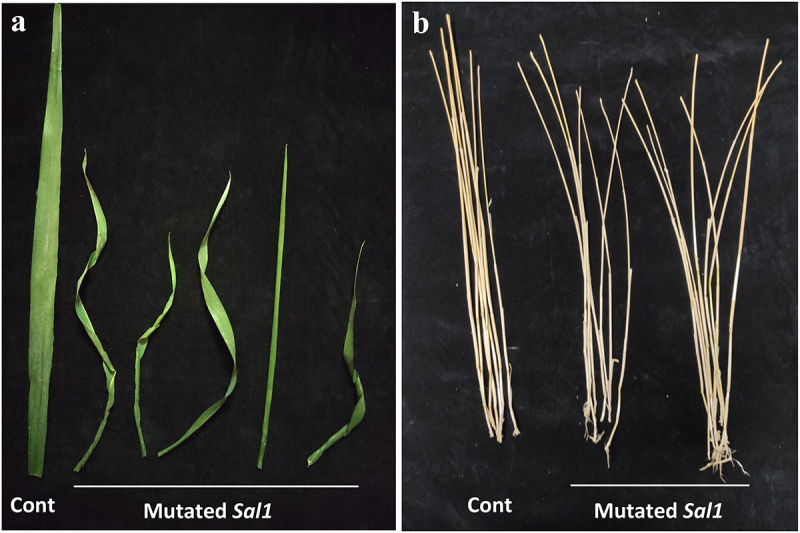
(a) Leaf of *TaSal1* M_2_ mutated lines with different rolled shapes. (b) Stems of *TaSal1* M_2_ mutated plants showing bending shape compared to the control.

The phenotypes of the Giza 168 *TaSal1* M_2_ mutants have been investigated in detail. Seedling germination and growth experiments were performed to test whether *TaSal1* knock-out lines grew differently than wild-type plants. This included using seedlings germinated on germination paper wetted with Hoagland’s solution, and we were unable to observe significant differences between the control plants and the *TaSal1* knockout lines. The obtained results showed that the loss of *TaSal1* function caused slight changes (rolling in the new leaf and bending in the stem), but in general it does not significantly alter the growth of seedlings (Figs. 3 and 4). Different parameters were measured including plant height, leaves number, number of tillers, and the number of reproductive tillers.

**Figure 5.**
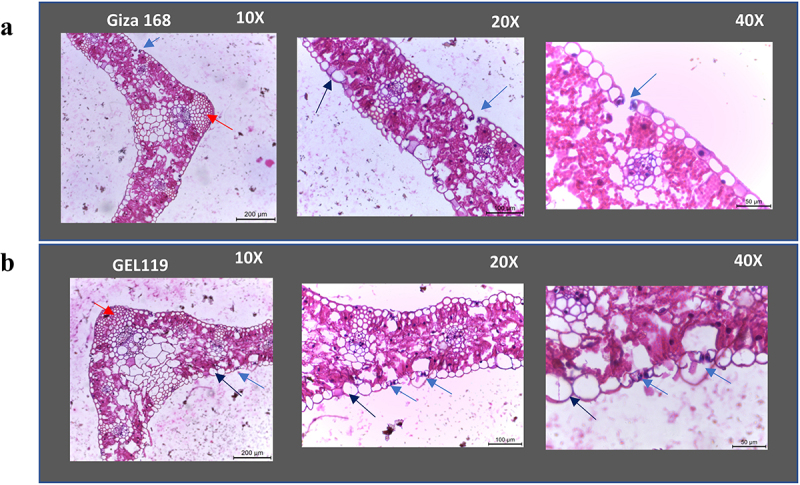
Transverse sections of leaves in control and M_2_ of Giza 168 cultivar. A: Control from non-transgenic plant showing open stomata B: mutated line GEL119 showing closed stomata (blue arrows). Large bulliform cells in leaf blade of the mutated line compared to the control (black arrows). Intensive sclerification around the vascular bundles in leaf bade of GEL119 (red arrows).

No significant differences were observed between the *TaSal1* mutant plants and the control in overall plant height, leaf number, tiller number, or reproductive tiller number between the mutants and control lines. Cross section of the first internode and the second internode showed slight differences in the shape compared to the control (Figure S4). Also, no significant differences were obtained in the length and width of first internode and the second internode (Figure S5). In addition, total chlorophyll measurement showed no significant differences between the mutated lines and the control (Figure S6). In general, there were no fundamental differences between the edited lines and control plants, the main differences were observed in the young leaves of *TaSal1* mutated plants as they showed different leaf roll as well as bended in the stems (Fig. 5).

The morpho-anatomical characterization of the young leaves of 5 M_2_ lines showed highly developed and bigger bulliform cells and intensive sclerification for the mutated lines compared to the control. In addition, M_2_ plants showed closed stomata in almost all parts of the leaf (Fig. 5). It worth to mention that in the old leaf of the mutated lines (no rolling) anatomical examination showed close stomata only in the area surrounding the core vein, while the control had open stomata in all parts of the leaves.

### Scan Electron Microscopy (SEM) Screening

**Figure 6.**
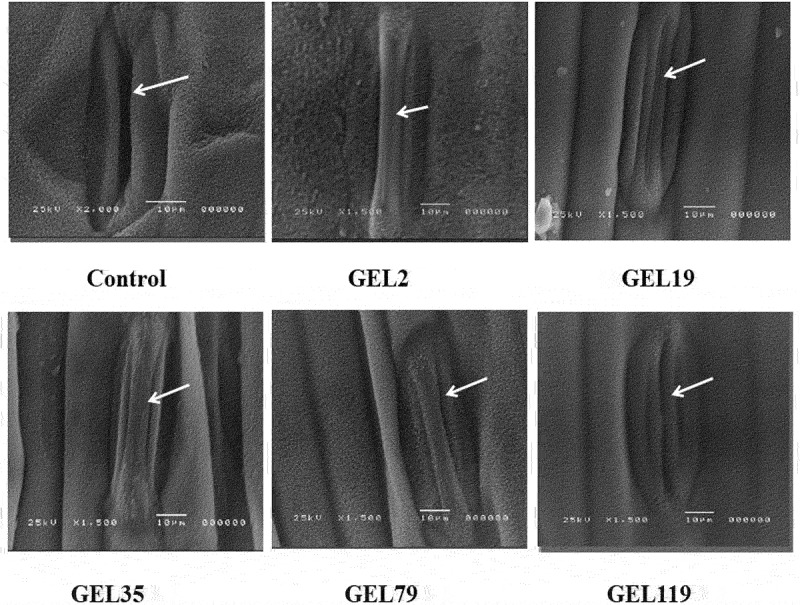
Scan electron microscopy scanning young leaves for Giza168 M_2_ lines stomata and control (non-mutated plant) from well-watered plants.

**Figure 7.**
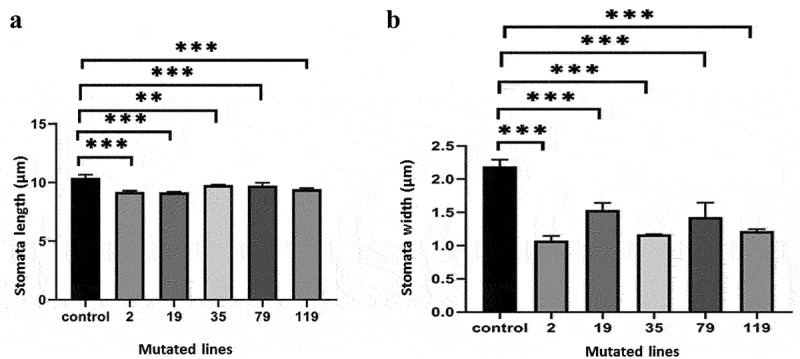
The length and the width of stomata guard cells of the 5 *TaSal1* mutated lines compared to the control from well-watered plants.

Analysis abaxial epidermis using the electron microscope scanning revealed a significant different between the young leaves of the 5 *TaSal1* mutated lines and the wild-type Giza 168 plant. Scanning electron microscope visualization showed a significant difference between the 5 *TaSal1* knock-out lines and the wild-type Giza-168 plants. The stomata appear closed in the mutants compared to the wild plants (Fig. 6) with difference in the measured width of the stomata opening between the *TaSal1* mutant plants and the wildtype controls (Fig. 7).

### Polyethylene Glycol (PEG) Drought Screening

**Figure 8.**
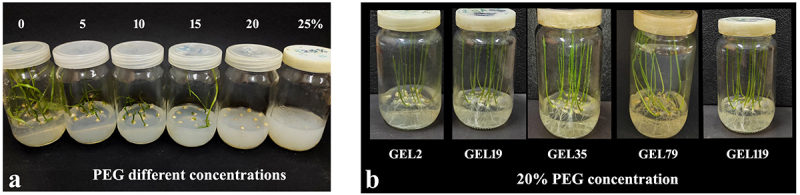
The seed germination on MS media supplemented with PEG. A: wild-type Giza168 seeds germinated on different concentration of PEG; B: control, E-02 and E-35 on MS media supplemented with 20% PEG.

To assess the ability of wheat mutated lines to grow under drought stress, M_2_ seeds of selected wheat Giza168 lines were sterilized and germinated on MS media supplemented with different concentrations (0, 5, 10, 15, 20 and 25%) of polyethylene glycol (PEG). The data indicated that, with increasing PEG concentration, the germination percentage, the shoot and root length differences between control (0% PEG) and plants that germinated at high PEG concentrations (20 and 25%) became apparent (Fig. 8a). The result showed that the germination percentage, shoot length, root length, fresh and dry weight were negatively affected under high PEG concentration (Table S5). The 15% PEG was the significant concentration for reduction the growth parameters while the concentration 20% was the critical concentration for seeds germination. Form this experiment, the 15% PEG concentration considered the maximum concentration for Giza168 germination. So, we choice the 15 and 20% PEG concentration to evaluate the ability of M_2_ Giza168 seeds germination. When compared with wild-type controls, *TaSal1* mutated wheat lines displayed better growth under high drought conditions (Fig. 8b). The mutated wheat lines showed no significant different performs under different PEG concentrations (Table 3).

**Table 3.** Mean performances of 3 wheat lines as factor (A) for germination %, shoot length, root length, fresh weight and dry weight affected by 3 different level of PEG Concentrations % (0, 15 and 20) as factor (B) and the effects of interaction among them (AB).

Wheat lines	Germination %				Shoot length (cm)				Root length (cm)				Fresh weight (g)				Dry weight (g)			
	0	15	20	Means A	0	15	20	Means A	0	15	20	Means A	0	15	20	Means A	0	15	20	Means A
GEL **2**	93.33	83.33	86.67	87.78	10.57	11.50	10.53	10.87	4.17	4.20	2.65	3.67	1.43	1.24	1.59	1.42	0.26	0.23	0.24	0.24
GEL **19**	90.00	90.00	80.00	86.67	9.60	10.91	10.34	10.57	4.10	4.14	3.87	4.04	1.40	1.63	0.77	1.27	0.20	0.44	0.08	0.24
GEL **35**	86.67	86.67	83.33	85.56	10.61	8.87	12.64	10.49	4.14	4.23	4.10	4.16	1.24	1.15	0.92	1.11	0.25	0.20	0.22	0.23
GEL **79**	90.00	83.33	86.67	86.67	10.57	11.50	10.53	9.79	4.13	4.07	3.14	3.78	1.21	1.55	1.36	1.37	0.27	0.23	0.36	0.29
GEL **119**	86.67	83.33	86.67	85.56	9.60	10.91	10.34	10.89	4.16	3.90	3.93	4.00	2.05	0.95	1.13	1.38	0.40	0.18	0.16	0.25
**Means B**	89.33	85.33	84.67		11.01	10.66	9.90		4.14^a^	4.11^a^	3.54^b^		1.47	1.31	1.16		0.28	0.26	0.21	0.28
***LSD _0.05_ A***	n.s				n.s				n.s.				n.s				n.s			
***LSD _0.05_ B***	n.s				n.s				0.45				n.s				n.s			
***LSD _0.05_ AB***	n.s				n.s				n.s.				n.s				n.s			

N.S. not significantly different at 0.05 level of significant (p > 0.05).

## Discussion

Abiotic stress conditions as in dehydration-inducing conditions are major limiting factors affecting wheat productivity and causing serious yield reduction worldwide.^20^ Plants respond to abiotic stress conditions through the activation of several signaling mechanisms to organize plant response.^21^ For plant species with a complex genome structure, as in wheat, genome editing could be the solution for genetic improvement.^24^ Genome editing could be the key for long-term challenge for molecular biology research, particularly for the complex genme of plants. In this work we developed an efficient regeneration and transformation systems for Giza 168 using mature and immature embryo explants. Also, we successfully generated plant edited plants with mutated *Sal1* genes in the complex wheat genome.

The *Sal1* gene, which encodes the enzyme 3’(2’),5’-bisphosphate nucleotidase, is usually located in the cellular organelles, and usually works by detoxifying the cellular PAP (monophosphate 3ʹphosphoadenosin 5’ phosphate via hydrolysis into adenosine monophosphate (AMP) and phosphate.^17^ It also considered the key player in PAP-dependent organelle-to-nucleus retrograde signaling.^22^ The PAP as a chloroplast stress retrograde signal accumulates during periods of drought and stress from salt and light.^17,25^ Cellular PAP levels are important for development and appropriate stress responses. It inhibits 5′- to 3′-exoribonuclease activity, and induces stress-responsive gene expression in the nucleus.^17^ SAL1-loss-of-function mutants results in PAP accumulation, which confer drought tolerance by enhancing drought-inducible chloroplast-to-nucleus stress signal and induce closure of stomata. SAL1 could be compensated for by a reduction of chloroplastidic but not cytosolic PAPS biosynthesis.^26^ Decrease the level of expression of SAL1 results in PAP accumulation and the associated cellular responses. in *Arabidopsis*, SAL1 plays an essential role in the regulation of stomatal closure and seed germination.^27^

In this context, *Sal1* genes (encodes the enzyme 3’(2’),5’-bisphosphate nucleotidase) were mutated to reduce the conversion of adenosine 3’,5’-bisphosphate (PAP) into adenosine monophosphate and inorganic phosphate. Based on the Chinese cultivar wheat genome, six *TaSal1* genes homeoforms were identified in six different chromosomes (4A1, 7A, 5A, 5B, 5D & 7D) and an addition were found in chromosome 4A of Bobwhite cultivar (4A2). Different Homeoforms specific primers were designed, and six copies of the *TaSal1* gene were assigned to Giza 168 genome, with the loss of one located on the 5A chromosome. Only five of the homeoforms were active as the 4A-1 gene has a stop codon. In the present study, we constructed a multiplex-CRISPR platform to disrupt the expression of the *TaSal1* gene in the genome of Giza168. Importantly, multiplex-CRISPR system has an efficiency of editing of several locations in the genome comparable to the corresponding single-function systems. The high efficiency of multiplex-CRISPR-Combo holds great promise for various applications in plants, such as reducing the generation time for editing several locations, as it is one major bottleneck for genome editing-based plant breeding.^28^ The multiplex CRISPR/Cas9 system we used in the current study is based on three conserved sgRNA sequences in all the 5 *TaSal1* genes. Furthermore, to increase the transformation efficiency, two constructs were prepared, the pCas2143 contains the tRNA-gRNAs expression cassettes together with the Cas9 gene, and the second contains the *Bar* gene as a plant selectable marker. Two explants’ materials were tested, mature and immature embryos, and out of 11047 regenerated plants, 120 plants were identified as Cas9 transgenics (4 derived from mature embryos, and 116 from immature embryos). Screening 41 M_1_ independent lines revealed that only five lines were mutated in all five *TaSal1* target genes.^29^

Previous work on Sal1 gene in other species indicated that Sal1 acts as a negative regulator of drought tolerance in *Arabidopsis*.^17^ The *Arabidopsis AtSAL1* responds to abiotic stresses, including salt, cold, drought, high light, and oxidative stresses.^24,28^ *Arabidopsis* mutants reduced in AtSAL1 activity were found to have enhanced the PAP levels while morphologically resembling 5′- to 3′- exoribonuclease loss-of-function mutants.^28,30^ These mutants exhibit increased tolerance toward various abiotic stressors and stomatal closure.^14,18,27,31,32^ This indicates that loss function in *AtSAL1* would enhance drought resistance in *Arabidopsis*, suggesting it would be a negative regulator of stress tolerance.^18,33^ The first plant reaction to drought is stomatal closure, caused by the loss of the turgor pressure of the two guard cells surrounding the stomal opening. ABA signals are one of the main signals that induce stomatal closure.^34^

Morphological studies of the M_2_ plants showed no significant differences in overall plant height, leaf number, tiller number, reproductive tiller number, and a cross section of the internode or the chlorophyll measurement between the mutant lines and the control. It was reported in rice that leaf rolling causes decrease in stomatal conductance and reduces water loss, especially under drought-stress conditions.^35^ The main differences between the mutated lines and the control were in the rolling of the young leaves and stem bending. However, previous work in *Arabidopsis* showed loose of function of SAL1 markedly affected the plant growth and development such as shorter petioles, wrinkled leaves, and anthocyanin.^36,37^ Stomata are the living interface responsible for >90% of plant water loss through transpiration. Stomata are important portals for gas and water exchange in plants and have a strong impact on the function associated with photosynthesis and transpiration. Stomata are vital to the existence of the plant as they control temperature and water-use efficiency (WUE). Environmental conditions greatly influence stomatal characteristics. Thus, stomata are a prospective target for improving drought tolerance by enhancing (WUE) in economically important cereals. Under dehydration-inducing conditions, plants increase their WUE by reducing stomatal aperture and thereby transpiration rate. However, under conditions of longed water deficit, plants frequently produce leaves with reduced maximum stomatal conductance resulting from altered SD and/or size.^38^ Also, plants tend to balance water, transpiration, photosynthesis, and WUE via changing stomata construction in order to prevent water content loss to adapt drought stress.^39^

Anatomical characteristics are excellent indicators of plant adaptation to environmental stresses like drought. In this work, we observed a precise stomata closure in the leaf of M_2_ plants using morpho-anatomical characterization and scanning electron microscope compared to the control. Also, more highly developed bulliform cells and intensive clarification was observed for M_2_ compared to the control. Also, highly developed bulliform cells and intensive sclerification were observed for M_2_ compared to the control. The highly developed and the larger size bulliform cells can minimize water loss from a plant surface,^40^ which can play a key role in improving degree of drought tolerance in plants subjected to drought stresses.

The results indicate that M_2_ plants were significantly more affected at the photosynthetic rate under drought stress than non-transgenics. Robertson et al.^41^ indicate that reducing stomatal density and aperture enhances WUE across multiple plant families, with minimal impact on the efficiency of both photosynthetic and carbon assimilation processes. In turn, the aforementioned stomatal modifications resulted in improve or stabilize crop yields under drought in commercial crop species. Loss function in *AtSAL1* enhanced drought resistance in *Arabidopsis*, suggesting that it work as a negative regulator for stress tolerance.^18^

One of the most popular approaches for developing plants with drought stress is to grow plants under high molecular weight osmotic substances, such as polyethylene glycol (PEG).^42^ These agents reduce the water potential of the culture medium in a way similar to soil drying, putting the plant under drought stress.^43^ In this investigation, seeds of the wild-type wheat Giza168 plants were short term drought-stressed by PEG 6000 with different concentrations (5, 10, 15, 20 and 25%) for 15 days to identify the maximum concentration for seed germination. Increasing the PEG concentration result in reduced germination percentage, shoot length, root length, fresh weight and dry weight with increased water stress level. Seeds had a very low germination on 15% PEG and were unable to grow over 15%. Growing M_2_ seeds of the five mutated lines under drought stresses (PEG 15 & 20%) showed healthy growing seedlings, compared to the control. The five blocked TaSal1 lines showed no significant differences between in germination ratio and seedling phenotypes. There were no significant differences between the five mutated lines. Adding PEG 6000 to culture media reduces the water potential of the medium that affects cell division, leading to reduced callus growth, which therefore affects the regeneration ability.^44^ PEG-6000 induced osmotic stress, may be associated with reduced cell division and elongation of cells during germination.^45^ A parallel decrease in plant regeneration with increasing *in vitro* osmotic stress has been reported for rice,^46^ wheat^47^ and sugarcane.^48^ Kafi et al.^49^ stated that as the water potential decreased germination percentage in lentil, root length, stem length, root dry weight, and stem dry weight decreased. The interaction effect of the PEG rates on the examined barley cultivars mentioned that PEG at 20% negatively affected the germination percentage.

From the obtained results, we hypothesize that the TaSAL1 protein has a key role as a negative regulator in the pathways enhancing drought tolerance. In addition, blocking the expression of TaSal1 in wheat did not have significant effects on the plant morphology. Also, we recommend using the multiplex-CRISPR system for mutating more than one location as it will open new door in plant genome engineering, metabolic engineering, and synthetic biology.

## Material and Methods

### Plant Material

Wheat Giza168 cultivar (*Triticum aestivum*) was used in this study that provided by Field Crops Research Institute (FCRI), Agricultural Research Center (ARC), Giza, Egypt. The pedigree of the Giza168 cultivar is MIL/BUC//Seri.

### *Identification of* TaSal1 *Genes from the Genome of Wheat Cv. Giza168*

Giza-168 seeds were surface-sterilized and planted into soil. At the three-leaf stage, the youngest leaf from 4 plants was harvested and genomic DNA extracted using the Gentra Puregene tissue kit (cat. no. 158667, Qiagen, USA). DNA from two plants was initially screened with gene-specific primer pairs using Phire Hot Start II DNA Polymerase (ThermoFisher Scientific, cat. no. F122L) to test for the presence of the known *TaSal1* genes.

Bioinformatics analysis of the publicly available hexaploid wheat genome sequence identified six wheat *TaSal1* gene homologs. These six genes encode proteins that share between 46% and 60% amino acid identities with the *Arabidopsis* Sal1 protein and thus they are potential functional orthologs of the *Arabidopsis* gene. Primers specific to amplify each of the six putative gene, in addition to additional gene found in chromosome 4A (*TaSal1* 4A-2 gene) of the genome of Bobwhite cultivar, were designed (Table S1). Genomic DNA from Giza 168 was used to amplify the *TaSal1* genes using Q5 high-fidelity DNA polymerase (New England Biolabs, M0491S). Bands were excised, gel-purified, and blunt-cloned into the pUC-Blunt vector at the *StuI* site. The ligations were transformed into competent *E. coli* cells and selected on LB media with 100ug/ml carbenicillin. Approximately 30–50 colonies grew on each plate; two colonies of each reaction and 8 colonies of the 5D reaction were grown overnight and plasmid DNA was isolated from the cultures. The presence of the expected insert was verified by restriction digests of the plasmid. One plasmid for each cloned PCR amplicon was DNA sequenced with M13F and Reverse primers. Amplified *TaSal1* genes were sequenced and submitted to the GenBank.

### *Construction of CRISPR/Cas9 Vector for* TaSal1 *Gene*

Conserved exon (coding) sequences in the wheat *TaSal1* homeoform genes were chosen as editing targets to ensure that small insertions or deletions would likely result in a frameshift or premature stop codon mutations, inactivating TaSal1 enzyme activity. The CRISPR MultiTargeter web tool^50^ was used to perform CRISPR guide RNA design, and from the list potential target sites, further bioinformatics analyses were used to rank the likelihood of the sequence being a specific and efficient targeting sequence within the wheat genome.^50–52^

Three gRNAs target exons 4, 5 and 7 of the wheat *TaSal1* genes were designed to knock-out the function these genes. The gRNAs are perfect matches to their targets, except for the 5′ region of *TaSal17D* has a single nucleotide mismatch (C instead of T) at position 14 of the sgRNA. Using gene synthesis, traditional restriction enzyme cloning and Golden Gate cloning methods, the CRISPR/Cas9 wheat transformation vector pCas2143 containing guide RNAs targeting *TaSal1* was constructed. Expression of the Cas9 gene is controlled by the maize *Ubiquitin 1* promoter/5′ intron and the nopaline synthase terminator. The Cas9 sequence is derived from pRGEB32.^53^ The gRNA expression cassette is a tandem array of tRNA and gRNA sequences assembled using pGTR as previously described by Xie et al.^53^ The constructed pCas2143 was designed to carry the *cas9* gene, the three *TaSal1* targeting guide RNAs (gRNA1, gRNA2 and gRNA3) flanked by transfer RNA (tRNA) spacers. The tRNA-gRNA assembly was inserted under the control of the switchgrass (*Panicum virgatum* L.) *Ubiquitin 1* promoter (PvUbi1) and *nopaline synthase* transcription terminator (nosT) (Figure. S1B). Following construction, the plasmid vector was validated via restriction enzyme digestion and sequencing with the primers shown in the table (S2).

### Wheat Transformation

In this study, the pCas2143 CRISPR plasmid in combination with the pAHC20 plasmid^54^ were used for co-transformation of wheat explants (Figure. S1). The pAHC20 construct carries the maize *Ubiquitin 1* (*Ubi1*) promoter and nos-terminator (NOSt) controlling the expression of the *bar* gene that confers resistance to the bialaphos herbicide and is used as a selectable marker to recover the transgenic wheat plants (Figure. S1A). The tissue culture and transformation were carried out as reported by Sivamani et al.^55^ for immature wheat embryos and according to Moghaieb et al.^56^ for mature wheat embryos. For microprojectile bombardment, the constructed vector DNA was delivered into the wheat explants using the He PDS/1000 Particle Delivery System. Bombardment was carried out with gold particle 1.0 µm in size. Each plate of wheat explant was bombarded once at a rupture pressure of 1100 psi and microcarrier travel distance of 6 cm with 5 µm of particle suspension mixture per bombardment. Preparation of gold particles and coating with plasmid DNA was carried out based on the manufacturer’s instructions (Bio-Rad, Hercules, CA, USA). Osmotic treatment of target tissue before and after bombardment was performed according to Vain et al.^57^ Bombarded tissue was placed on the same culture medium supplemented with 5 mg/l bialaphos for 4 weeks at 25°C in the dark. Bialaphos-resistant calli were transferred to regeneration medium (MS medium containing 2% sucrose, 0.15 mg/l thidiazuron and 1 mg/l bialaphos) for 2–3 weeks at 25°C under a 16 h photoperiod. After 2 weeks, regenerated shoots were transferred to rooting medium (MS supplemented with 2.5 mg/L IBA and 2 mg/l bialaphos) for 2–4 weeks at 25°C under the above light conditions. Plantlets were transferred from rooting medium to greenhouse potting mix and were covered with beakers for the first few days after transplantation to prevent desiccation. Greenhouse day: night temperatures were 25:19°C under a 16 h photoperiod with supplemental lights to provide 150 mmol m^_,1_2^ light intensity.

### Screening of Transformed Plants Using Polymerase Chain Reaction (PCR)

In order to confirm the stable integration of *cas9* gene into the wheat plant genome, the putative transgenic plantlets at M_0_ were analyzed by PCR using two different primers for amplified *Cas9* gene, and the gRNAs sequence (Table S3). DNA samples were extracted from both the transformed and non-transformed (control) plantlets utilizing plant Simply^TM^ Genomic DNA Extraction Kit (GeneDireX® cat.no.SN025-0100). The PCR reactions were performed in a 20 μl volume including 2 μl of extracted DNA (50 ng/μL), 1 μl of each primer (2 μM/μl), 10 μl GeneDireX® One PCR^TM^ (cat.no. MB203-0050) master mix and 6 μl sterilized double-distilled water. PCR program was optimized at 94°C for 5 min initial denaturation, followed by 35 cycles of denaturation at 94° C for 1 min, primer annealing at 55°C or 56°C for 1 min (Table S3) and primer elongation at 72°C for 2 min. The final extension was 5 min at 72°C. All the PCR results were then loaded in 1.5% agarose gels stained with ethidium bromide for electrophoresis and visualized by a Biometra UV star transilluminator.

### CRISPR-induced Mutations Screening

#### Cleaved Amplified Polymorphic Sequence (CAPS) Detection

For mutation screening at the molecular level, M_1_ mutated lines were utilized for genomic DNA extraction. DNA samples were extracted utilizing plant Simply^TM^ Genomic DNA Extraction Kit (GeneDireX® cat.no.SN025-0100). Fifteen pairs of primers were design to identify small insertions or deletions in the targeted gRNAs for the different sites of the *TaSal1* genes (Table S4). Mainly mutations were first screened at the 3ʹCRISPR target sites in all 5 of the *TaSal1* genes by restriction cutting with *Xcm*I, which overlap with the targeted cut site (which is 3 bp 5’ to the PAM. Identified lines with mutation on the 3ʹCRISPR target sites were sequenced for the three target sites (5’, middle and 3’). In addition, two other recognition sites for *Bat*XI and *Bbs*I enzymes may be used for screening large deletion mutation on middle and 5ʹCRISPR target sites, respectively. The PCR cleanup was done utilizing Wizard®SV Gel and PCR clean-up system (Promega, USA, cat. no. A9280), followed by sequencing at Macrogen Company, Korea.

#### Morphological Screening

Induced indel *TaSal1* mutants as well as the control were analyzed for phenotypic variations in M_2_ generation. The first internode length, the first internode width, the second internode length and the second internode width were used to evaluate morphological change^58^ in the nonmutated Giza168 plant and *TaSal1* mutated lines. Total chlorophyll content in leaves was measured from 3 weeks old plants using SPAD units through monitoring of chlorophyll meter (SPAD- 501).^59^

#### Leaf Anatomy Measurements

One cm piece from the M_2_ lines leaf center along the midrib was taken and for leaf sheath one cm piece from the nodal region surrounding the stem was selected. The material was preserved in FAA (formalin acetic alcohol) solution for fixation, which contained v/v formalin 5%, acetic acid 10%, ethyl alcohol 50% and distilled water 35% for at least 48 hrs.^37^ Materials were embedded in paraffin wax of melting point 56°C. Leica RM2125 microtome was used to section them at a thickness of 20 micron, double stained with safranin and fast green, cleared in xylene, and finally mounted in Canada balsam. Slides were analyzed microscopically by Leica ICC50 HD cam and photo-micrographed.

#### Scanning Electron Microscopy (SEM) Screening

To prepare samples for SEM, pieces (5 × 5 mm) of flag leaves and wheat ear parts were removed with a sharp knife and fixed in cool 4% glutaraldehyde (pH 6.8). The fixed samples were then washed with 0.1 M phosphate buffer to remove glutaraldehyde. Samples were dehydrated by a series of dehydration solutions (10, 30, 50, 70, 80, 90, and 95% ethanol, each for 10–20 min, and then two rounds of 100% ethanol, each for 20–30 min). After dehydration, the samples were treated stepwise for 15 min in mixtures (75% ethanol + 25% isoamyl acetate, 50% ethanol + 50% isoamyl acetate, 25% ethanol + 75% isoamyl acetate) and finally were soaked in 100% isoamyl acetate for 30–40 min. After critical point drying with CO_2_, samples were pasted on the sample stage, and coated with gold in a sputter coater. Samples were observed and photographed with a JSM-5200 Scanning Electron Microscope. According to the actual ratio of obtained images, the size were measured and calculated under a single view (2,230 µm^2^). The length and width of the stomata were calculated using JSM-5200 Scanning Electron Microscope. All samples were taken simultaneously and under the same conditions where the plants were well-watered irrigated.

#### Polyethylene Glycol (PEG) Screening

To evaluate the ability of wheat Giza168 cultivar for drought tolerance, MS media supplemented with different concentrations (0, 5, 10, 15, 20 and 25%) of PEG were used. Giza168 seeds were sterilized with ethanol 70% for 1 min then with 20% Clorox (hypochlorite sodium) for 20 min and washed three time with sterilized distend water. Ten seeds were plated on MS medium with different concentrations of PEG. After 15 days, the germination percentage, shoot length and root length were recorded. The M_2_ seeds of mutated Giza168 wheat lines were germinated on MS media supplemented with different concentrations (0, 15 and 20%). Ten seeds per jar and 5 jars for each treatment. The experiment was performed in triplicate.

## Statistical Analysis

A one-way analysis of variance (ANOVA) was carried out using Graph Pad Prism 7 for Windows 10 computer software package and Dunnett’s multiple comparisons test at α = 0.05.

## Supplementary Material

Supplemental Material
