# Supplementary material for: Multiplex CRISPR/Cas9-mediated genome editing to address drought tolerance in wheat
Source: GM Crops Food. 2022 Oct 6;16(1):1–17. doi: 10.1080/21645698.2022.2120313 (PMC11702957; doi:10.1080/21645698.2022.2120313)
Supplement: Supplemental Material [file KGMC_A_2120313_SM1053.zip › Supplemantary_Tables.docx]

Table S1: Nucleotide sequence of primer pairs used for amplifying *Sal*1 gene

| **Sal1 location** | **Primer Sequences**  **5’→3’** | **Annealing temp. (**°C) | **Expected size (bp)** |
| --- | --- | --- | --- |
| **4A-1** | TTCGTTCTCTCAACTTCCCG  CCTGTGTCACGGTCAAGAAA | 65 | 1828 |
| **4A-2** | TGCCTGTATCTGTTGTTTGGT  TCAGTTGAGTTCATCGTTTCG | 63 | 1900 |
| **5A** | TCCTGGACAGACTTCAGCAG  AGTGCTTGTGTTTGCTGCTT | 67 | 2974 |
| **5B** | AGTTTCTTGAGAGGAGGCCA  TCCTGGACAGACTTCAGCAG | 67 | 901 |
| **5D** | GGTTCATTTTCTATGGTGGCCGAA  TCCTGGACAGACTTCAGCAG | 68 | 2664 |
| **7A** | TGGTACATGCTCACTCTGTA  GTGTCACGATCAAGATATCTGC | 63 | 2339 |
| **7A** | TGGTACATGCTCACTCTGTA  GTGTCACGATCAAGATATCTGC | 63 | 2339 |

Table S2: Sequence of designed primers used for sequencing the putative Sal1 genes in Giza168

| **Primer Number** | **Sequence 5’→3’** | |
| --- | --- | --- |
| Seq-8 | TGTGGAATTGTGAGCGGATA | |
| Seq-685 | CAGGAGCCTCAAGAGTGGAC |  |
| Seq-938 | TCAGGGTTATTGTCTCATGAGCGG |  |
| Seq-1149 | GCAGTTCGAAGGAGATAGAACC |  |
| Seq-1150 | GGACACCTACGACGACGACC |  |
| Seq-1151 | TCCGTGGAAATCTCCGGCG |  |
| Seq-1152 | GACCAAGGCCGAGAGAGGC |  |
| Seq-1153 | CCTCTGCCGGCGAACTGC |  |
| Seq-1242 | GAGGCTTGTGTTACTGCTAG |  |

**Table S3:** **Primer sequences used to validate transgenic Giza-168 plants.**

| **Primer Name** | **Sequences (5′ → 3′)** | **Expected amplicon Size** (**bp**) | **Annealing Temperature** |
| --- | --- | --- | --- |
| Cas9-F | AGACAGTGAAGGTGGTGGAC | 142 | 56°C |
| Cas9-R | CTCGTCGTACTTAGTGTTCATC |  |  |
| gRNAs-region-F | AAGGAAGTTTAAGGACTCAGAAGACTTGAGAA | 620 | 55°C |
| gRNAs-region -R | CGTGGCAGGGTACTATTCTACC |  |  |

**Table S4.** **Primer sequences used for screening for *Sal1* targeted mutations.**

| **No.** | **Primer name** | **Sequences (5^’^→3^’^)** | **Annealing Temp.** | **Expected product bp** | **Chromosome** | **gRNA** |
| --- | --- | --- | --- | --- | --- | --- |
| **1** | 4A2-F1 | AATAAGGAAATAACTTCTGGC | 50 | 256 | 4A2 | 5’ |
|  | 4A2-R1 | CAGAGAGGATAGCTTCCTTG |  |  |  |  |
| **2** | 4A2-F2 | GATCAGTTAGCTGGCAAGATC | 57 | 326 |  | Middle |
|  | 4A2-R2 | AGCTACCACTGAGGTTACTTG |  |  |  |  |
| **3** | 4A2-F3 | GCTTAGGCTATATCCATCGT | 52 | 258 |  | 3’ |
|  | 4A2-R3 | TCAGTTGAGTTCATCGTTTCG |  |  |  |  |
| **4** | 7A-F1 | CCATTCAGGTGATAATTTTA | 49 | 475 | 7A | 5’ |
|  | 7A-R1 | AATTGGCGGAAAAACCAGACA |  |  |  |  |
| **5** | 7A-F2 | TCAGTTAGCTGGCAAGATTGT | 49 | 436 |  | Middle |
|  | 7A-R2 | AATAAAAGAGATTGAAAGTGA |  |  |  |  |
| **6** | 7A-F3 | AGCGGAGGTTTGATCTTGATT | 57 | 219 |  | 3’ |
|  | 7A-R3 | CCGGCTGCATGATCCCATAC |  |  |  |  |
| **7** | 5B-F1  5B-R1 | TAGGATCTCAAGTATTGGTAAGTCTCGT  GGACCTCCTTCAGACTTCCCA | 54 | 2071 bp | 5B | 5’ |
| **8** | 5B-F2 | TTTCTTGAGAGGAGGCCAATA | 54 | 158 |  | Middle |
|  | 5B-R2 | TAGCTGAAAAGAGGGCCCCT |  |  |  |  |
| **9** | 5B-F3 | TAGATTAGTGTTTGTACCATT | 51 | 443 |  | 3’ |
|  | 5B-R3 | CTGGGGAACACACGAAAACGT |  |  |  |  |
| **10** | 5D –F1 | AGAGGTAATTCACTTGTGTTG | 48 | 206 | 5D | 5’ |
|  | 5D-R1 | ATACCTTCCTTAGATAAAGT |  |  |  |  |
| **11** | 5D –F2 | CTGAAAAACAATGATATGCAA | 48 | 214 |  | Middle |
|  | 5D-R2 | TTGAGGTTGCTTATTGATGC |  |  |  |  |
| **12** | 5D –F3 | CCGTTTTAGTAGAAACTAATA | 49 | 171 |  | 3’ |
|  | 5D-R3 | CATGATCCCATATCTTTTCCT |  |  |  |  |
| **13** | 7D-F1 | TTCTTTTCATACATGTAGGG | 54 | 385 | 7D | 5’ |
|  | 7D-R1 | GCTCACCCTTTAGTCCCATCG |  |  |  |  |
| **14** | 7D-F2 | GCTCATGTTGTTACTATCTCT | 54 | 232 |  | Middle |
|  | 7D-R2 | CAGCTGAAAAGAGGGCCCCA |  |  |  |  |
| **15** | 7D-F3 | GGCTAACTTTCATTACAGATC | 56 | 347 |  | 3’ |
|  | 7D-R3 | CCTGTGACGACAATTGAC |  |  |  |  |

**Table S5: The effect of different PEG concentrations on Giza168 wild plant seed germination, shoot length, root length, fresh and dry weight.**

| **PEG concentrations** | **Studied traits** | | | | |
| --- | --- | --- | --- | --- | --- |
|  | **Germination %** | **Av. Shoot length (cm)** | **Av. Root length (cm)** | **Fresh weight (g)** | **Dry weight (g)** |
| **0%** | 80.00 ^a^ | 11.32 ^a^ | 3.90 ^a^ | 1.46 ^a^ | 0.22 ^a^ |
| **5%** | 75.00 ^ab^ | 7.59 ^a^ | 2.10 ^b^ | 0.80 ^b^ | 0.12 ^b^ |
| **10%** | 60.00 ^b^ | 7.81 ^a^ | 1.75 ^b^ | 0.53 ^b^ | 0.11 ^b^ |
| **15%** | 5.00 ^c^ | 2.08 ^b^ | 0.84 ^bc^ | 0.06 ^c^ | 0.01 ^c^ |
| **20%** | 0.00 ^c^ | 0.00 ^b^ | 0.00 ^c^ | 0.00 ^c^ | 0.00 ^c^ |
| **25%** | 0.00 ^c^ | 0.00 ^b^ | 0.00 ^c^ | 0.00 ^c^ | 0.00 ^c^ |
| **L.S.D _0.05_** | **16.5** | **3.63** | **1.34** | **0.44** | **0.08** |


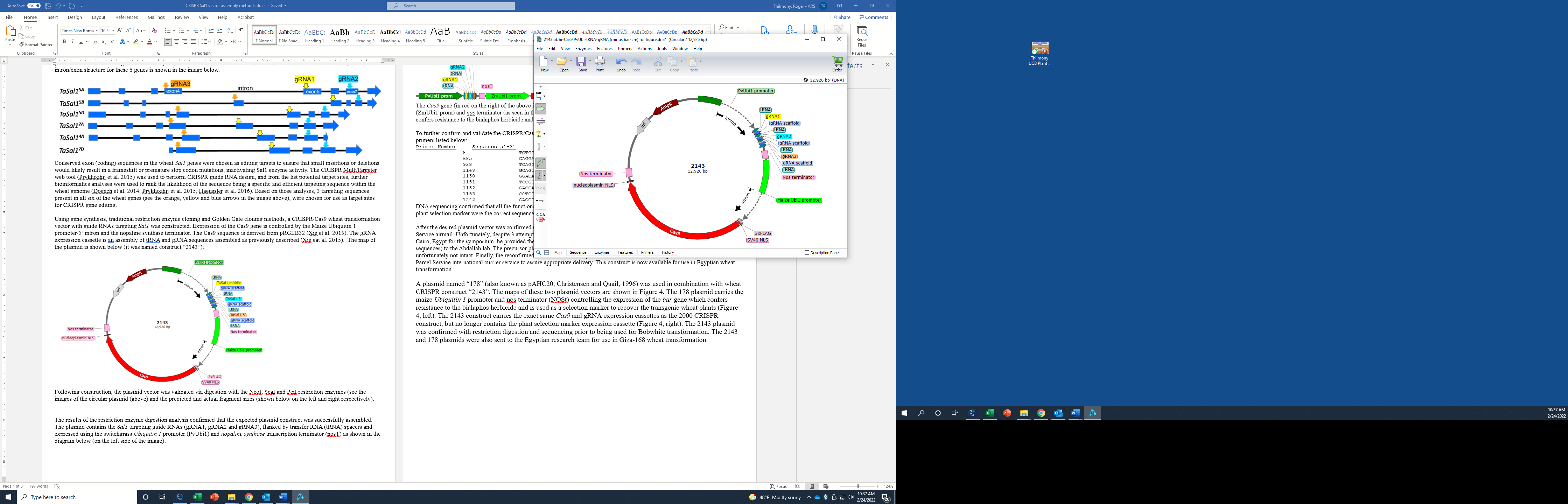

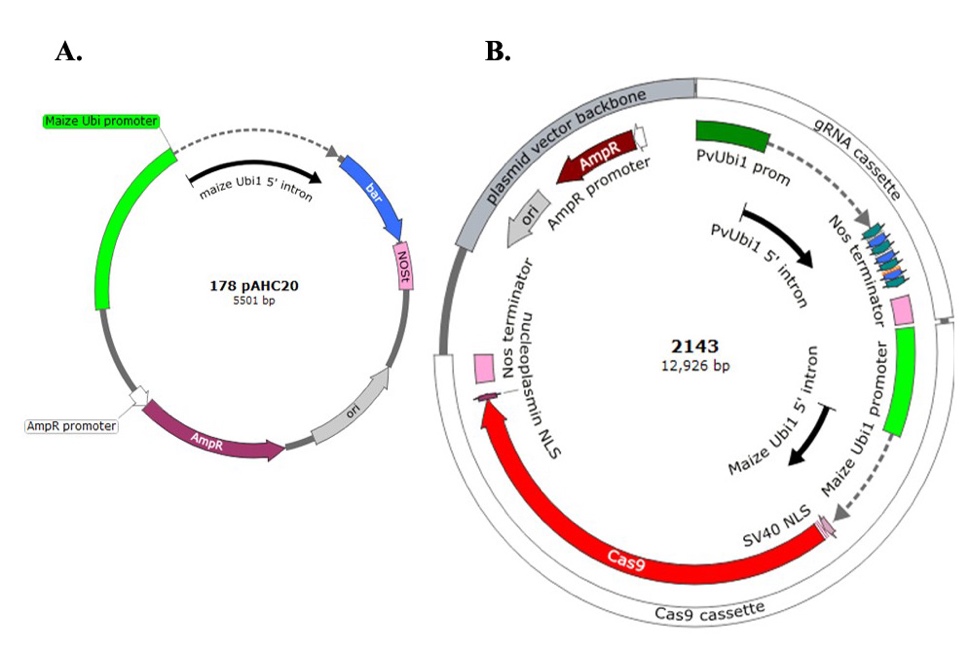


**B.**

**A.**

pCas2143

12,926 bp

pAHC20

5501 bp

**Fig. S1:** The two plasmids used for co-transformation of wheat explants. A: the pAHC20 plasmid containing the bar selectable marker and B: the pCas2143 constructed plasmid containing the Cas9 gene and the three sgRNA sequences under the PvUbi1 promoter.


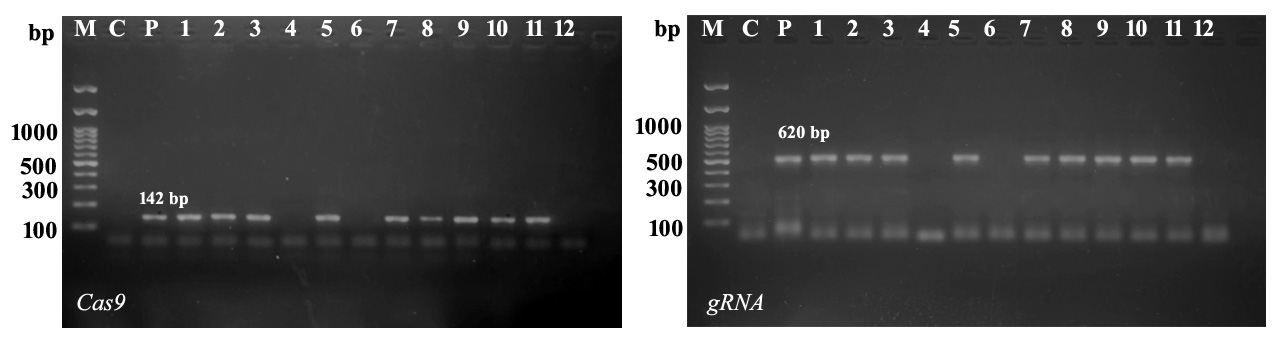


Fig. S2. Detection of the *Cas9* and *gRNA* specific primers in transformed Giza-168 wheat plants. Lane M is the 100 bp DNA ladder. Lane C is the negative control (non-transformed wheat), lane P is the positive control (plasmid 2134) and lanes 1-12 are candidate transformed Giza-168 wheat lines.


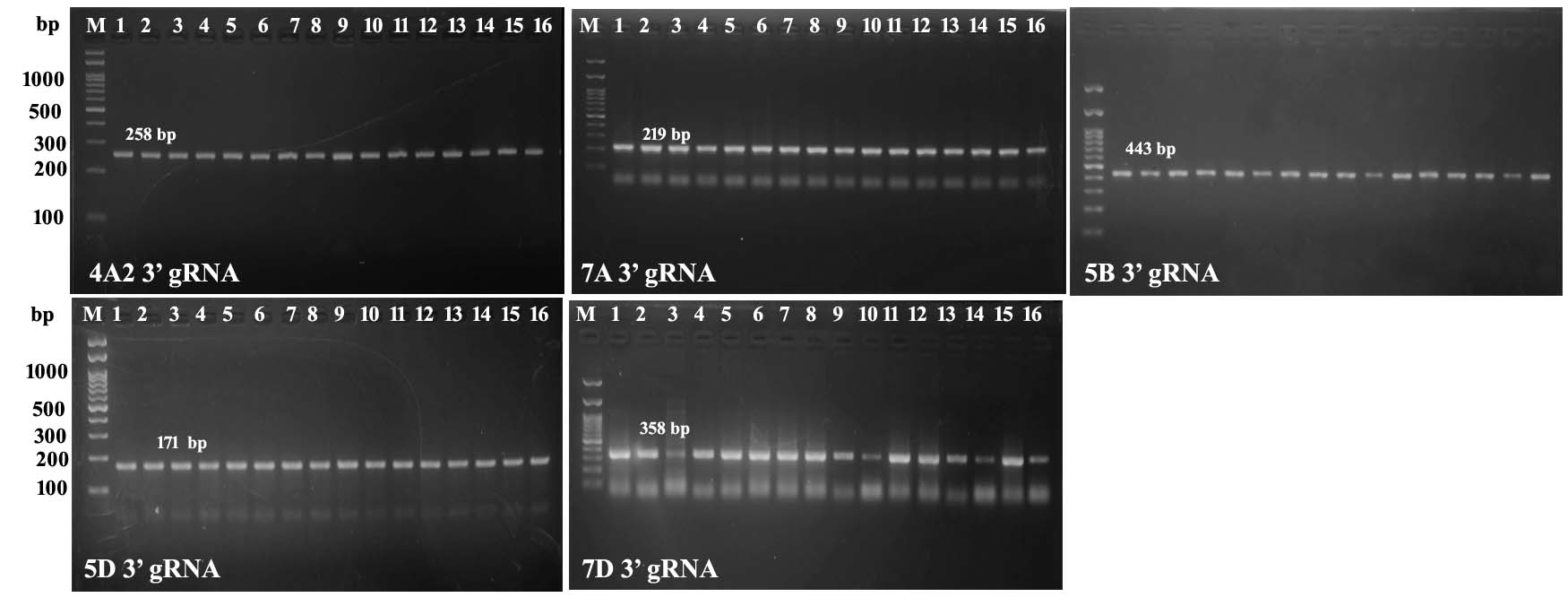

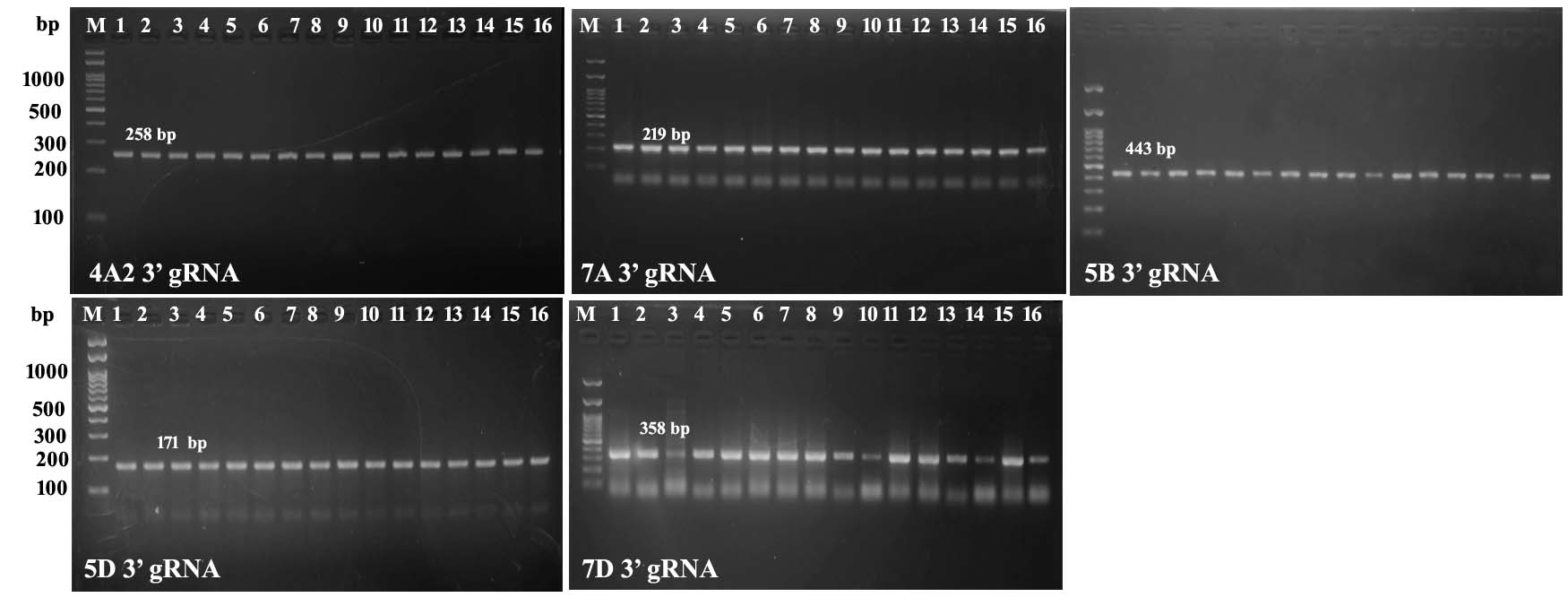


Fig. S3. The amplified products for the Giza-168 *Sal1* genes using specific primers for each targeting 3’ of target CRISPR sites for the 5 *Sal1* genes. M is 100 bp DNA markers.


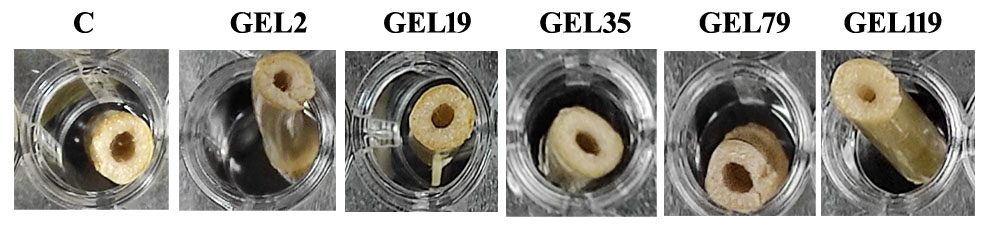


Fig. S4: Cross section of the M_2_ first internode for the stem of the five wheat Giza168 genome edited lines (GEL2, GEL19, GEL35, GEL79 and GEL119) and control (non-mutated plant).

**
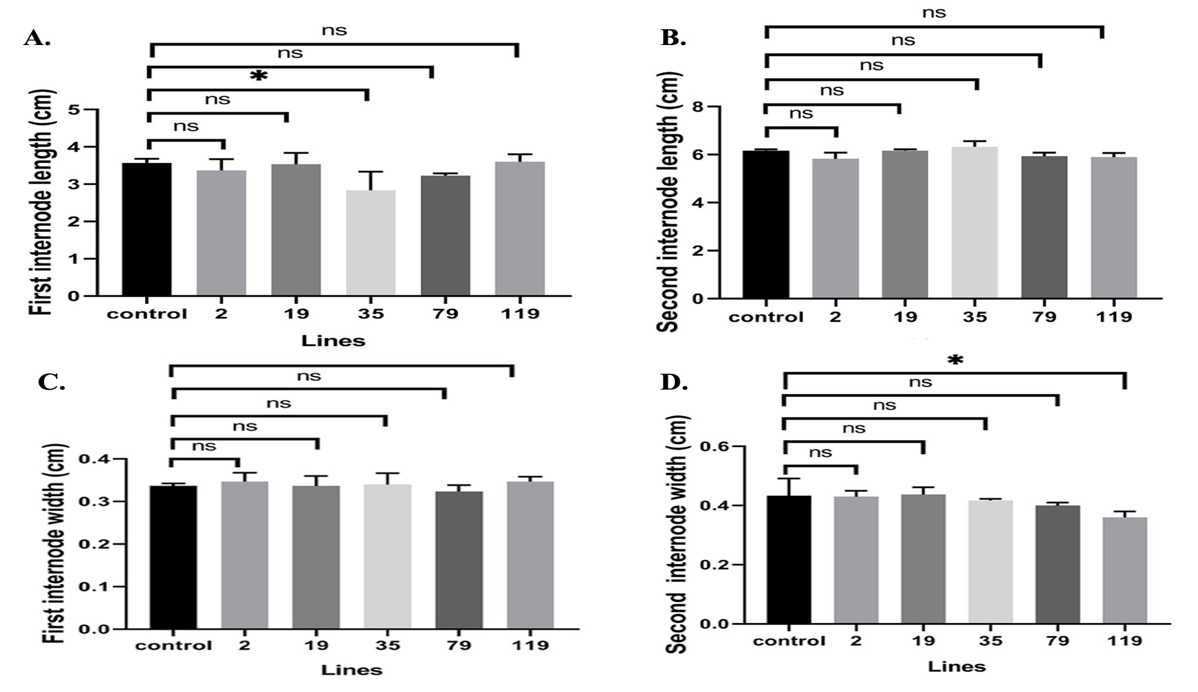
**

Fig. S5: Growth measurement of the M_2_ first and the second internode length and width of the five wheat Giza168 genome edited lines (GEL2, GEL19, GEL 35, GEL 79 and GEL 119) and control (non-mutated plant) showing no or low significant (p<0.01).

**
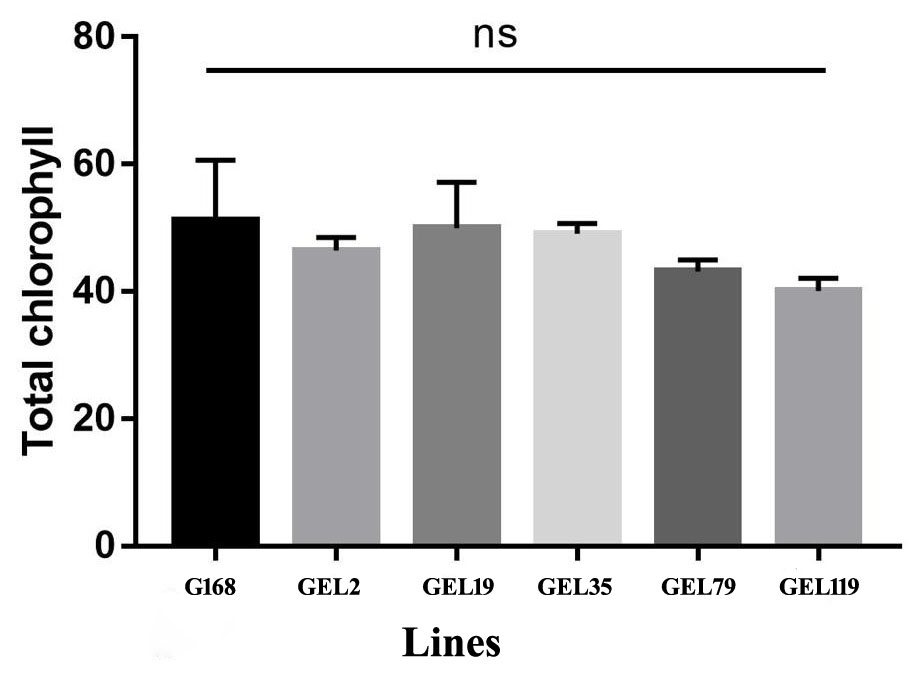
**

**Fig. S6: Total chlorophyll well-watered mutants and control at M_2_ stage showing no significant differences between them.**
