# Supplementary figures and images for: Multiplex CRISPR/Cas9-mediated genome editing to address drought tolerance in wheat
Source: GM Crops Food. 2022 Oct 6;16(1):1–17. doi: 10.1080/21645698.2022.2120313 (PMC11702957; doi:10.1080/21645698.2022.2120313)

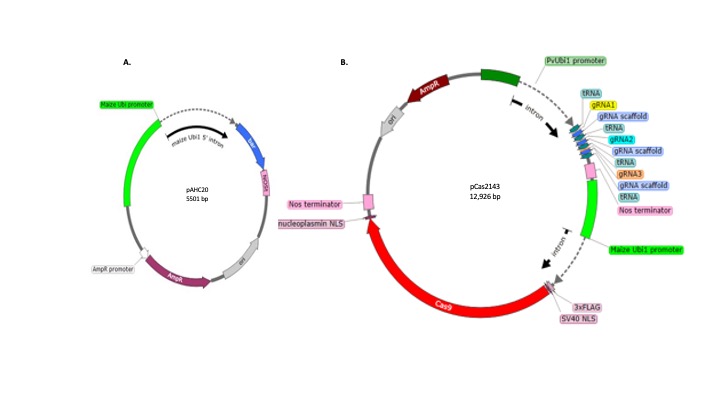

Supplement: Supplemental Material [file KGMC_A_2120313_SM1053.zip › S1.jpeg]

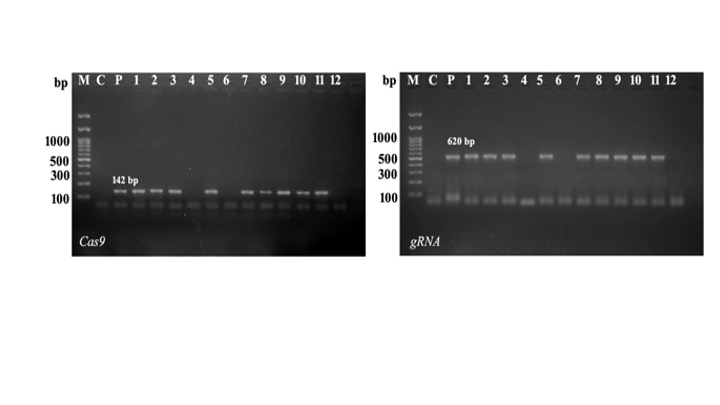

Supplement: Supplemental Material [file KGMC_A_2120313_SM1053.zip › S2.jpeg]

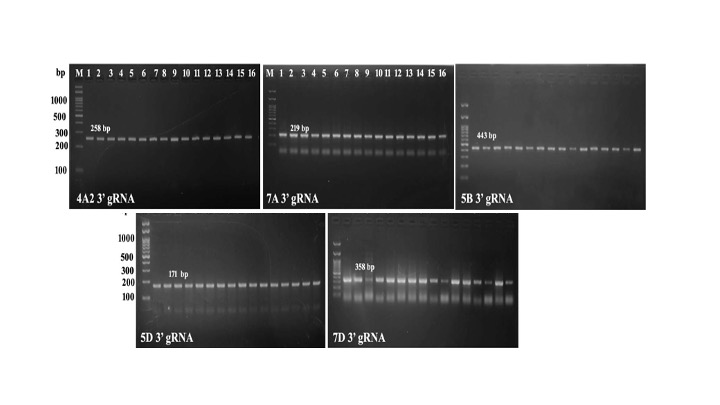

Supplement: Supplemental Material [file KGMC_A_2120313_SM1053.zip › S3.jpeg]

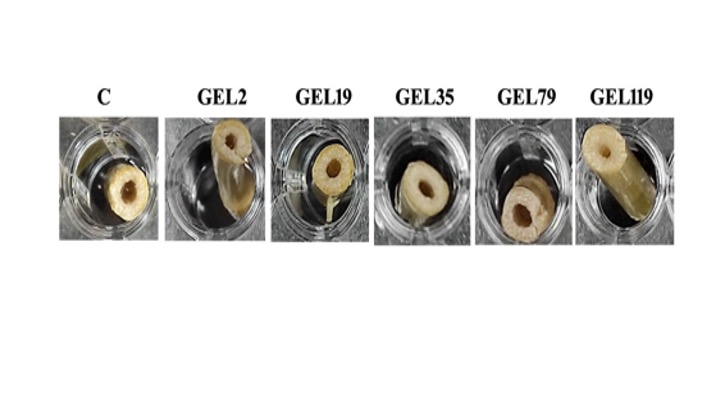

Supplement: Supplemental Material [file KGMC_A_2120313_SM1053.zip › S4.jpeg]

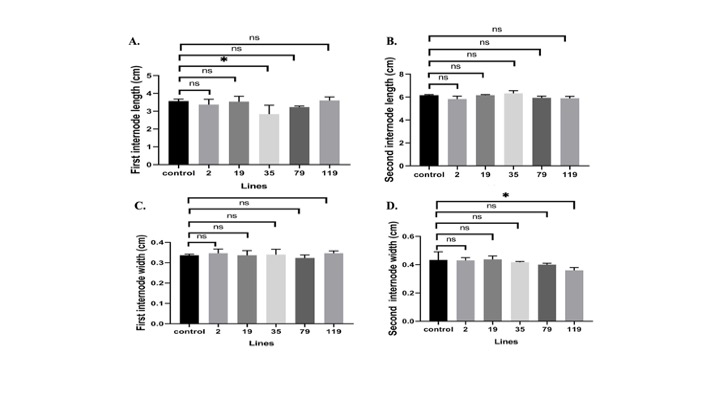

Supplement: Supplemental Material [file KGMC_A_2120313_SM1053.zip › S5.jpeg]

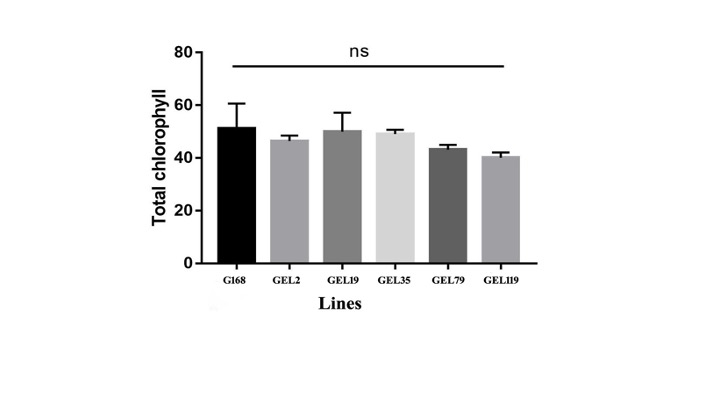

Supplement: Supplemental Material [file KGMC_A_2120313_SM1053.zip › S6.jpeg]
